# Book Reviews

**Published:** 1803-04-01

**Authors:** 


### CRITICAL ANALYSIS

OF THE

### RECENT PUBLICATIONS

ON THE

### DIFFERENT BRANCHES OF PHYSIC, SURGERY, AND MEDICAL PHILOSOPHY.

---

*Some Observations on the present Epidemic Catarrhal Fever or Influenza, chiefly in relation to its Mode of Treatment. To which are subjoined, Historical Abstracts concerning the Catarrhal Fevers of* 1762, 1775, *and* 1782. By RICHARD PEARSON, M. D. 8vo. pp. 26.

THE Catarrhal Fever, or *Influenza,* which has been for some weeks very generally diffused over this metropolis and its environs, and is now spreading to distant parts of the island, is a subject of considerable interest to medical practitioners, and still more so to those who examine with a philosophic eye into the obscure causes of general epidemics. The disease, to which the term *Influenza* is now irrevocably fixed, has appeared, at intervals, varying over almost every part of Europe; and as it has seldom been of a nature to spread alarm and terror, it has excited all the attention due to so interesting an epidemic from the enquiring physician. The great similarity, amounting almost to identity, of nature between these several epidemics is fully established, but we have still to search for their remote and predisposing cause; and many important questions relating to their nature and character still remain unanswered. In the present instance, it is much too soon to attempt the general history of the epidemic, and the author has therefore confined himself chiefly to a simple relation of the prevailing symptoms, and (what is of immediate consequence) to the mode of treatment which has been found the most successful. He announces his intention of pursuing his inquiries into the subject; which we trust will also be done by every intelligent practitioner in the kingdom. As our Journal may probably reach several parts of the island before the arrival of the epidemic, we shall give, in the Author's words, the whole of his description of its attack.

"The Catarrhal Fever, or Influenza, which is now spreading itself over the whole metropolis, and will probably soon make its way to every part of the kingdom, first shewed itself here towards the end of the last month (February), when a damp and mild state of the atmosphere had succeeded to severe cold. This again has been followed by frost and keen easterly winds during the first part of the present month, (March). Like all similar preceding epidemics, the present Influenza has exhibited various degrees of morbid affection; having been, in some instances, so slight as not to incapacitate persons from continuing their ordinary occupations and pursuits, and scarcely to require the aid of medicine; whilst, in others, the attack has been of such a malignant nature as to endanger and even destroy life.

"The following is its most frequent mode of attack. After some alternations of chilliness and heat, the patient is seized with a heaviness or pain of the head, with sneezing, wateriness of the eyes, hoarseness, and cough. These symptoms come on in the order here stated. In the course of a few hours the head-ach increases, the skin becomes hot, with a pain in the back and limbs, or transitory stitches across the chest. The tongue is white; the pulse quick or frequent, and for the most part soft. There is more or less of sickness at the stomach, and sometimes vomiting. The bowels are generally costive, and considerable uneasiness, often amounting to great pain, is felt in some part of the abdomen. By the second or third night, the cough and fever become greatly aggravated. The former, viz. the cough, is strong, incessant, sometimes dry, but generally accompanied, even at its first coming on, with an expectoration of thin, sharp mucus; the latter, viz. the fever, is attended with increased heat, and with extreme restlessness and anxiety. There is also some confusion of the head. At this time the pulse is often from 100 to 120. In the morning there is a considerable remission of the febrile symptoms; but the cough still continues urgent, and the patient complains of excessive languor and dejection of spirits.

After the fourth or fifth day, where early perspirations have come on, or sufficient evacuations have been procured by the bowels, the fever declines; and although the cough continues, the expectoration is more free, the sputum being of a thicker consistence and milder quality. The urine, which before was high coloured and clear, now becomes turbid, or throws down a sediment. In other instances, the cough goes off without any remarkable degree of expectoration. The lassitude and depression of spirits with restless nights, harass the patient for many days after the decline of fever; which, indeed, in several instances, does not entirely go off after the fifth day, but becomes intermittent, the patient feeling himself worse every other day.

"Such is the most common form of this epidemic. Its modifications, however, as we have before observed, are extremely numerous, so that in some there is a violent head-ach with little catarrhal affection; in others, a sore throat; in others, a peripneumonic condition; and in others, a disordered state of the stomach and bowels."

The above description is sufficiently clear and accurate, if meant to exhibit the genuine form of the epidemic unmixed, (as may be inferred) with the symptoms of any other disease or morbid tendency which may happen to be present at the time of attack. The swelling of the eye-lid and inflammation of the conjunctiva might have have been mentioned more particularly, as they have, in several instances, been the most marked, and sometimes the only symptoms of the epidemic.

The Author then proceeds to the method of cure, which he introduces by the very judicious observation, that, "as it is the fever which constitutes the essence of the disease, our first attention should be directed to it, and not to the cough, (except when it is accompanied with a true peripneumony); otherwise, by prescribing only for one of its symptoms, we shall make but little impression upon the general morbid affection." Pectoral remedies are not at first to be resorted to, but we should use emetics and mercurial and antimonial cathartics. For the latter purpose, he recommends calomel, joined with about half its weight of pulvis antimonialis, assisted, if requisite, by a solution of some neutral salt. The calomel and antimony seldom fail speedily to produce general diaphoresis, which relieves the symptoms of general fever, but not the cough. This latter, and the dyspnœa, require the application of a blister, to which may be joined the use of aqua ammoniæ acetatæ and ether. After the bowels have been opened, opiates afford relief, but they must be given in very sparing doses. A solution of cream of tartar forms a very proper drink, which may be taken without reserve. In the decline of the disorder, when the cough is still troublesome, squills and ether may be used with advantage, but oily medicines and the pectoral emulsion are improper. The languor, dejection of spirits, and debility left by this epidemic, are so considerable, that, after the first or second day, all attempts to keep up the sweating process should be avoided, the patient should sit out of bed. and the room kept cool and well ventilated. During convalescence, which is often very slow, the author recommends infusions of the bitters combined with natron, but the Peruvian bark and the mineral acids, he observes, are of no avail, and even do harm. The diet should be thin, mild, and mostly vegetable; broths should be avoided, as they keep up an unsalutary perspiration, and increase the head-ach, nausea, and languor.

This is the plan laid down by the author, which is judicious and temperate. In the essential particulars it agrees with the general practice that has been adopted in this metropolis, but in some points it differs so much, that we think more attention might have been paid to this subject. If the author had considered the oily and pectoral emulsions as merely insignificant auxiliaries, little or no notice of them would have been necessary; but as they have actually been employed in London with the most unsparing profusion, an absolute condemnation of these trifling remedies might have been accompanied with some remarks. The same of bark, and the mineral acids, though they have been less used than the former. Blisters have been found so highly beneficial in many instances, that they well deserved a warmer recommendation.

The present epidemic has proved fatal to a considerable number of persons, when combined with pulmonic inflammation or habitual

 asthma.

asthma. The treatment of these delicate cases is referred to the general treatment of peripneumony, but without that discrimination which in the present period would be peculiarly interesting.

On the whole, we can only consider this pamphlet as a sketch, traced out with a hasty pencil, and if generally correct, somewhat deficient in the filling up.

Extracts from the most authentic accounts of the former epidemic catarrhs, or influenzas, are subjoined, which shew in a very satisfactory manner their identity with the present epidemic, and with each other.

---

*Cases of Cancer, with Observations on the Use of Carbonate of Lime in that Disease; by* Edward Kentish, M. D. Newcastle upon Tyne, 1802. pp. 48, 8vo.

When the matter of fact contained in these pages is stripped of the profusion of words, of irrelevant matter, and of needless amplification, with which it is invested, it will be found to amount to little more than this, viz. that the author had under his care two cases of perfectly well marked cancer, of the open or ulcerated species, in both of which the treatment that he adopted was the same, and certainly judicious; and to which he employed powdered chalk as the principal topical application, with a success that would be admirable, if a general average could be drawn from two cases, since one terminated in a compleat cure, and the other fatally.

The cases themselves are interesting, and described with apparent fidelity and accuracy. We shall give the author's account of the mode of treatment. In the successful case both breasts were affected, the right was ulcerated; and at the same time the left was swelled and painful, and a thin ichorous discharge oozed out from around the nipple, which threatened rapid ulceration. With a view of equalizing the action of the glandular system, as the author expresses it, and preventing the engorgement which had already taken place in various glands terminating in the death of those parts, he was induced to use the vapour-bath, the effect of which, in discussing hard tumours, he would seem to refer to the solvent power of steam upon inanimate vegetable and animal matter, combined with the local stimulus of heat inducing an equal action on the part affected;—a jumble of ideas, of which he promises an explanation on some future occasion! The carbonate of lime (or chalk) he employed to the ulcerated cancer at the time when the secretion was fully established, and very abundant. The idea of this application was suggested to the author by its well known success in several cases of burns, where the secreting surface is extensive, the discharge profuse, and the process of cicatrization very tardy. As this is the most interesting part of the present treatise, we shall give the author's own explanation of the rationale of the effect of this remedy. "The carbonate of lime was applied half an inch thick, morning and evening, in bare contact with the sore, sore. This covering repressed the growth of fungus in the central part of the wound, and encouraged the formation of cuticle at the edges. It appeared as if the lime, uniting with the secretion of the cellular membrane, formed a pearly coating similar to the lining of oyster-shells, or probably like shelling in the case of birds. Nor does it seem improbable that the process of skinning, like that of shelling, may be considerably assisted by the addition of lime, either as a carbonate or a phosphate. Birds, at the laying season, as canaries, are incapable of supplying a sufficient quantity of calcareous matter, unless they have a supply of it with their food. Hens, I am informed, if prevented from this supply, lay eggs without shells. Marine animals secrete much calcareous matter; the annual renewal of their shell proves this. The whole bony fabric of animals has lime for its basis, and the secretion in some cases of fracture is very abundant, as is also the want of this principle in some cases of mollities ossium. If lime make so great a part of the animal as the bony system, and if the want of it prevent the purposes of Nature from being carried into effect, as is observed in birds, it is highly probable that in some old obstinate ulcerations, benefit might be derived from giving this principle internally, as well as from its external use." What are we to understand from this medley of absurd and false analogy? Does the Author really mean to assert that calcareous earth actually enters into the composition of the cuticle, as it does into that of bone? Or, does he suppose that the process of skinning is no more than plastering up with animal mucilage and chalk? He might as well talk of *skinning* a wall by white-wash, which is composed of animal size and chalk. The unlearned reader might infer from our Author's explanation of the process of skinning, that marine animals produce their shells by moistening their skin with cellular secretion, and then rubbing themselves against a chalk rock; or, that birds acquire their covering by a sort of natural tarring and feathering.

In the very next sentence however, the author gives a very sensible explanation of the use of chalk, in a few words, namely "that it absorbs the redundant secretion in a more perfect manner than the substance which has been so long in use among surgeons, I mean, the lint or charpie, with which it is the custom to fill all wounds. It also corrects the fœtor arising from ill conditioned wounds."

The progress of this case is interesting; we shall give it in the Author's words: "The deep foul ulcer which presented itself when the mammary glands were thrown off, left a very considerable void. This was filled up to the level of the adjoining cuticle with very fine levigated and prepared creta. The fœtor of the discharge became less, the matter was also of a better consistence, and in smaller quantity. In clearing away the incrusted edges of the sore with a spatula, after having taken away several coatings, I had the extreme pleasure of perceiving the shelly pellicle forming upon the cellular membrane, at an acute angle with the cuticle. The wound was was dressed once a day; and at the end of a month I first began to observe this appearance. From such an effort, I became extremely anxious to second the endeavours of Nature. Some fungusses, which appeared to obstruct this process, yielded to the reiterated applications of alumen ustum, over which the creta, as usual, was applied. There still remained some points where the ulcers continued rebellious, notwithstanding the application of the alumen. To give them a new surface, which I was in hopes I should be enabled to cicatrize, I applied, by means of a probe armed with lint and moistened, some of Plunkett's powder, made according to the recipe in the Pharmacopœia Chirurgia. By this means I was enabled just to touch the points of the sore which appeared to me to require it; I afterwards put on the powdered creta as before. The arsenical powder prevented the growth of fungus."

The author very judiciously compleated the cure of the successful case by the application of straps of adhesive plaster, in the method which he is pleased to call Mr. Baynton's, but which in similar cases had been employed, long before that gentleman called the attention of the Faculty to its more extensive use.

In so terrible and hopeless a malady as confirmed cancer, every rational attempt at cure, or relief, even every plausible project, should meet with attention. The solitary successful case of Dr. Ewart, where carbonic acid was employed, was followed up by repeated experiments, in which unfortunately the sanguine hopes that might have been entertained, were frustrated; equal attention should be paid to the present proposal, and we earnestly wish that it may meet with better success.

---

*An Account of the Galvanic Experiments performed by* John Aldini, *Professor of Experimental Philosophy in the University of Bologna, &c. on the Body of a Malefactor executed at Newgate, January* 17, 1803. pp. 34, 8vo.

Galvanism is every day offering so many new objects to our research, that we have no hesitation in pronouncing it the most curious subject of inquiry that is now engaging the attention of the lovers of natural philosophy. The experiments lately performed in this town by Prof. Aldini, must be fresh in the memory of those who were gratified with a sight of these infinitely interesting phenomena; and to those who were not so fortunate, this short narration will be perused with interest, and may suggest a variety of new and untried objects of inquiry. The subject of the experiments was a malefactor executed at Newgate, on the morning of the 17th of January last. The body was exposed a whole hour in a temperature of about 30°, after which it was delivered to the College of Surgeons, in pursuance of the usual sentence of the law, and was transferred to Prof. Aldini, who with the assistance of Mr. Keate, Mr. Carpue, Mr. Cuthbertson, and other able men, subjected it to the following experiments, the Galvanic power being in all of them supplied supplied by three troughs, combined together, each of which contained forty plates of zinc and as many of copper; what the interposed fluid was, is not mentioned.

1. One arc being applied to the mouth and another to the ear, (wetted with a solution of common salt) the jaw immediately began to quiver, the adjoining muscles were horribly contorted, and the left eye actually opened.

2. On applying the arc to both ears, a motion of the head was manifested, all the muscles of the face became convulsed, and the lips and eye-lids were evidently affected. The action was increased by making one extremity of the arc to communicate with the nostrils and the other with the ear.

3. On applying the conductors to the ear and to the rectum, such violent muscular contractions were excited, as almost to give the appearance of re-animation.

4. Volatile alkali was first applied to the nostrils and mouth, but without the least sensible action; but Galvanism immediately excited violent action. This however was still more increased by uniting both of these stimuli, and applying them together.

5. The fibres of the biceps flexor cubiti were laid bare, and one arc was applied to them, whilst the other remained in the ear. This produced violent convulsions of all the muscles of the arm.

6. One arc remaining in the ear, and the other being applied to an incision in the wrist, among the small filaments of the nerves and cellular membrane, a very strong action of the muscles of the fore-arm and hand was perceived. In these two last experiments the natural moisture of the part was sufficient to conduct the Galvanic influence without the intervention of salt water.

7. The short muscles of the thumb were dissected, and on applying the conductor to them, a forcible effort to clench the hand was induced.

8. The effect of other powerful stimulants, caustic ammonia, concentrated sulphuric acid, and the mechanical stimulus of pricking the fibres with the point of the scalpel, were tried by way of comparison with Galvanism, but without producing the smallest effect.

9. The thorax and pericardium being opened, and the heart exposed in situ, the conducting arc was applied to the *ventricles*, first upon its surface, then in the substance of its fibres, then upon the carneæ columnæ, and the septum ventriculorum, and, lastly, in the course of the nerves by the coronary arteries, but without being followed by the slightest contraction or muscular motion.

10. The arc was then conveyed to the *right auricle* and the appendix auricularis, and considerable contraction was immediately produced, even without the intervention of salt water. In the *left auricle* however, scarcely any motion was excited.

11. Conductors being applied from the spinal marrow to the fibres of the biceps flexor cubiti, the glutæus maximus, and the gasterocnemius, separately, no considerable action in the muscles of the arm and leg was produced.

12. One

12. One of the conducting arcs being applied to the spinal marrow, and the other to the sciatic nerve, (which was exposed between the great trochanter and the tuberosity of the ischium, and dissected out from its sheath) no contraction whatever ensued in the muscles. But the conductor being removed from the nerve to the adjacent muscular fibres and cellular membrane, as strong an action was manifested as in the former experiments.

13. By making the arc to communicate with the sciatic nerve, and the gastrocnemius muscle, a very feeble action was produced in the latter.

14. Conductors being applied from the sciatic to the peronæal nerve, scarcely any motion was excited in the muscles.

15. The sciatic nerve being divided about the middle of the thigh, and conductors being applied from the biceps flexor cruris to the gastrocnemius, there ensued a violent contraction of both.

These are the principal circumstances of the interesting experiments which are here related; and the reader will readily concur in the leading inference which Prof. Aldini deduces from them, namely, that the power of Galvanism, as a stimulant, is stronger than any mechanical action whatever, and that hence it affords very powerful means of resuscitation in cases of suspended animation under common circumstances, perhaps superior to any that are usually employed. This consideration may serve to give this study additional interest to those who are not satisfied with any object of philosophical pursuit, without discovering in it an immediate prospect of benefit to mankind. The physiologist however will not fail to perceive the variety of curious questions for future inquiry, involved in the singular unsusceptibility of the ventricles of the heart to a stimulus which powerfully affects every other muscle of the body, and in the habitude of muscle with this influence compared with that of nerve and medulla spinalis.

From the circumstance of the rapid exhaustion of the power of the troughs, and the extent of active metallic surface requisite to produce the Galvanic action in sufficient intensity to exhibit the above phenomena, the Author infers the probability that the method of coating nerves with metallic surface, as first practised by Galvani, (on frogs and cold-blooded animals) serves merely to conduct the fluid pre-existent in the animal system; whereas, with Volta's Galvanic batteries, the muscles are excited to action by the influence of the apparatus itself.

Hence again, the Galvanic action, as excited by the batteries, may be conveyed to a greater number of points, and consequently be rendered more powerful in producing muscular contraction, if at the same time the assistance of metallic coatings be resorted to, and the two methods of Galvanic excitement be combined.

The Author, however, does not enter into this subject, but reserves it for two complete and interesting publications, which he announces for subscription, (*at Cuthell and Martin's, Middle Row, Holborn*) one of which will contain an Account of the late Improvements provements in Galvanism, with the different experiments lately performed by himself (Aldini) in Bologna, Paris, and London; and the other will be an Historical View of the Origin and Progress of Animal Electricity and Galvanism, with an Account of the Life and Writings of Galvani.

One of the principal difficulties in instituting comparative experiments on the operation of Galvanism, is the perpetually varying energy of the pile or battery; a difficulty which it is the less easy to remove, as the test and measure of this influence in exciting muscular contraction is equally variable, according to the original force of vital action, or remaining susceptibility to stimuli which the animal organ possesses and retains.

A short account of the appearances on dissection are added by Mr. Carpue, with which we shall conclude our Review of this short but interesting publication. The blood in the head was not extravasated, but several vessels were prodigiously swelled, and the lungs were entirely deprived of air. There was a great inflammation of the intestines, and the bladder was fully distended with urine. In general, upon viewing the body, it appeared that death had been immediately produced by a real suffocation.

---

*Observations on the Epidemical Diseases now prevailing in London, with their Divisions, Method of Treatment, Prevention, &c.; by* Robert Hooper, M. D. *Resident Physician to the St. Mary le Bone Infirmary, &c.* London, 1803. pp. 43, 8vo.

The author employs the term *epidemic* as an aggregate, composed in the present instance of several epidemical diseases, in general considerably distinct from each other, but possessing many symptoms in common. This perhaps would be a just method of considering the present *Influenza*, if the distinction between the diseases could be fairly made out; for it is highly probable, that at a time when any epidemic is very generally prevalent, all the diseases common to the same season of the year should have an unusual tendency to spread abroad, and by assuming certain leading symptoms, should acquire a mutual resemblance.

This view of the subject pre-supposes that the peculiar epidemic is not contagious, but that it is produced by the state of the atmosphere, or some exciting cause to which every person is equally exposed, an opinion which the author adopts in its fullest extent. He does not attempt, however, an explanation of the immediate exciting cause of the prevailing epidemic; the remote causes which he gives, are the usual ones of catarrh and peripneumony, and do not apply with more peculiar force to the circumstances of the two last months, than to the same season of other years that have not been visited with any general epidemic.

The author then proceeds to consider "the symptoms which accompany most of the prevailing epidemical diseases." These are described with great accuracy, and in these the reader will find all that is generally generally understood by the term *Influenza*, as now applied in common conversation.

The first of these is *Pain of the Head*, a very characteristic symptom, which has seldom if ever been absent. This is described sometimes as a sensation like the opening and shutting of the skull, sometimes a mere throbbing pain in one or both temples; at other times the whole head feels as if it had been beaten. This pain is constant, increases towards night, often almost to delirium. In many instances it is the only symptom of the epidemic. It is relieved by leeches to the temples, blisters behind the ears, and in relaxed habits, by volatile and stimulating lotions.

*Pain in the Chest.* This has been found to be either of the acute pneumonic kind, increased by full inspiration and attended with dry cough and fever; or to be acute and lancinating, often shifting its place, shooting to the back and shoulders, and apparently confined to the muscles of the thorax. The former species required the vigorous antiphlogistic practice suited to peripneumony; the latter the more moderate and mixed practice employed in pneumonia notha.

*Pains of the Limbs;* a symptom which has been almost peculiar, and as characteristic of the present Influenza as the head-ach. These pains have a great general resemblance to rheumatism, are attended with a quick pulse and a white rheumatic tongue, but scarcely in a single instance has the limb been swelled. In coughing, this pain shoots down from the head and neck along the course of the spine and large nerves.

*Dry and contracted Skin.* This is only at the beginning of the disorder, for perspiration has in general been remarkably easy to excite by antimonials and diaphoretics.

*Pulse* frequent, but in general soft; and otherwise natural, except true peripneumony has been present. In this respect it much resembles the pulse of peripneumonia notha.

*Tongue,* uniform in its appearance, and very characteristic. From the beginning to the termination of the disease the tongue has been white in the centre, red at the edges, and studded with very florid papillæ.

*State of the Bowels;* almost always costive.

*Catarrhal Symptoms;* constant coughing, slight sneezing, soreness of the nostrils, and great defluxion from the nostrils and mouth.

*Prostration of Strength;* very considerable throughout the disease, and embarrassing to the practitioner, on account of its interfering with the vigorous depleting practice necessary to the peripneumony when present. Fainting has been a very common and very alarming accident, which has occurred in most of the cases that have terminated fatally.

*Vertigo, Delirium, Coma;* these symptoms have not been common; sometimes the acuteness of the head-ach has produced some degree of vertigo, but comatose delirium has only attended the true pneumonic cases, and in general was the precursor of a fatal termination.

*Vomiting,*

*Vomiting*, has occurred spontaneously in a few instances, but with little relief to the head-ach. The author ascribes it to peritoneal inflammation of the stomach.

The above is a short abstract of the author's account of the leading symptoms of the epidemic, symptoms which he gives as the peculiar character of the epidemic abstractedly considered. He next proceeds to the species of epidemical diseases now prevalent, of which he enumerates the following:

1st. *Peripneumonia vera*, in its genuine character, requiring a cautious but decided antiphlogistic and depleting practice. 2d. *Peripneumonia notha, or rheumatism of the chest and its viscera*, principally distinguished from the former by the constant shifting of the pains. This has been relieved by blisters, stimulating liniments, ether and Dover's powder internally, and light opiates, together with calomel to relieve the bowels. 3d. *Catarrhus, or cold from contagion*, in which all the ordinary symptoms of catarrh were present, but with much more than usual severity. It has been treated with lac amygdalæ and nitre, syrup of poppies, light diaphoretics, and opiates. In this form, the disorder has been almost an ephemeral. 4th. *Febris rheumatica, or true rheumatic fever*, beginning with severe pain over every part of the body without exception, swelling of the face and eyes, and a white tongue with red edges, but rarely swelling of the limb. The greater number who have suffered under this form of the epidemic were men. For the cure, blood-letting has sometimes been employed with advantage; antimonials, salines, aperients and opiates, always useful.

The Author adds the appearances on dissection, which, as might be expected, have varied considerably, since the fatal events have arisen from several distinct causes.

Such are the general contents of this very interesting Treatise, which we earnestly recommend to all our Readers, as containing much important information concerning the epidemic which has been so generally prevalent in this metropolis for several weeks.—As a nosological history, much might be objected as to the form under which the Author has introduced his observations; and we cannot think that he has succeeded in his attempts to mark the limits of (what, perhaps, is in its nature illimitable) the morbid symptoms peculiar to, and characteristic of, the prevailing epidemic, and those of the specific diseases to which these symptoms appear to be attached. The matter of fact, however, contained in this Treatise is highly valuable, and the practice laid down is judicious and discriminative; nor is this of small importance in the present emergency, since the concurring testimony of all who are professionally concerned in attending the sick, or in doing the last offices to the deceased, puts it out of doubt that the mortality in London and the adjacent districts, has been and still is unusually great; and that to a great proportion of it, something of a common cause or character of disease may be ascribed.

